# The value of effective public tuberculosis treatment: an analysis of opportunity costs associated with multidrug resistant tuberculosis in Latvia

**DOI:** 10.1186/1478-7547-11-9

**Published:** 2013-04-17

**Authors:** Thaddeus L Miller, Andra Cirule, Fernando A Wilson, Timothy H Holtz, Vija Riekstina, Kevin P Cain, Patrick K Moonan, Vaira Leimane

**Affiliations:** 1 University of North Texas Health Science Center at Fort Worth, School of Public Health, Fort Worth, TX, USA; 2 State Agency Infectology Center of Latvia, Riga, Latvia; 3 Division of HIV/AIDS Prevention, Centers for Disease Control and Prevention, Atlanta, GA, USA; 4 Division of Tuberculosis Elimination, Centers for Disease Control and Prevention, Atlanta, GA, USA; 5 World Health Organization Collaborative Center for Research and Training in Management of Multidrug-resistant Tuberculosis, State Agency Infectology Center of Latvia, Riga, Latvia

**Keywords:** Tuberculosis cost, Health economics, Cost analysis, Evidence based policy

## Abstract

**Background:**

A challenge to effective protection against tuberculosis is to sustain expensive and complex treatment public programs. Potential consequences of program failure include acquired drug resistance, poor patient outcomes, and potentially much higher system costs, however. In contrast, effective efforts have value illustrated by impacts they prevent. We compared the healthcare costs and treatment outcomes among multidrug-resistant tuberculosis (MDR-TB) and non MDR-TB patients in Latvia to identify benefits or costs associated with both.

**Methods:**

We measured and compared costs, healthcare utilization, and outcomes for patients who began treatment through Latvia’s TB control program in 2002 using multivariate regression analysis and negative binomial regression.

**Results:**

We analyzed data for 92 MDR-TB and 54 non MDR-TB patients. Most (67%) MDR-TB patients had history of prior tuberculosis treatment. MDR-TB was associated with lower cure rates (71% vs. 91%) and greater resource utilization. MDR-TB treatment cost almost $20,000 more than non MDR-TB.

**Conclusion:**

Up to 2/3 of MDR-TB treated in our sample was preventable at a potential savings of over $1.3 million in healthcare resources as well as substantial individual health.

## Background

Tuberculosis control poses significant challenges despite existing highly successful, relatively inexpensive treatment [1,2]. A principal challenge is duration—usually no less than 6 months—of chemotherapy, which contributes to non-adherence, treatment failure, and—if not completed—the acquisition of anti-tuberculosis drug resistance [1-3]. Consistent and thorough management of tuberculosis by health authorities using the World Health Organization’s widely adopted DOTS strategy is key to avoiding treatment failure and its consequences [4-9]. Evidence links treatment failure and its consequences to insufficient management of active tuberculosis. Still, robust tuberculosis control programs are often not priority for public investment [10,11].

Effective and sustained tuberculosis control and achievement of elimination goals is possible through public health system efforts and the population-based infrastructure they provide. Many health needs exist, however, and policy makers often lack evidence to objectively identify priority among them. Cost and outcome analysis can provide useful evidence to guide decision makers toward supporting the “most effective medical care affordable” [12].

It is increasingly important to quantify the economic value of effective tuberculosis control in order to justify, prioritize, and sustain public investments toward it [13,14]. Health, economic, and other losses may be especially substantial in drug-resistant tuberculosis. Cure of drug-resistant, and especially MDR-TB is less likely, and requires more toxic and expensive treatment [3,15,16]. Yet acquired drug resistance can be prevented. Consistent use of best practices in tuberculosis management, such as prompt diagnosis, including drug susceptibility testing and direct observation of therapy, may be expensive yet cheaper than the alternative.

Budget constraints can be acute in regions such as Eastern Europe as they move beyond the economic and political turmoil left after the dissolution of the former Soviet Union (FSU). Latvia is one such nation, and like many of its neighbors has a high burden of tuberculosis and a significant incidence of MDR-TB (Table 1). Latvia reported MDR-TB prevalence of 14.3% and 10% in 2008 and 2009 respectively [10]. This is in comparison to a median prevalence of 9% (range: 1.6% - 22.3%) for 26 other high burden countries [17].

**Table 1:** Comparison of tuberculosis morbidity and mortality between various countries in 2009

	**Latvia**	**Estonia**	**Lithuania**	**Romania**	**Ukraine**	**USA**
**Population (millions)**	2.205	1.283	3.536	21.905	45.135	313.232
**Life expectancy (yrs)**	72.68	73.33	75.34	73.98	68.58	78.37
**Death rate (deaths/1,000)**	13.6	13.55	11.33	11.81	15.74	8.38
**TB incidence**	1,100	450	2,600	27,000	59,000	14,000
**TB rate per 100,000 pop**	49.89	35.07	73.53	123.26	130.72	4.47
**MDR-TB incidence**	131	86	322		3,482	
New cases (% of total MDR)	83 (63%)	54 (63%)	114 (35%)		1,437 (41%)	
Previously treated (% of total MDR)	48 (37%)	32 (37%)	208 (65%)		2,045 (59%)	
**Number of TB cases with +HIV (%)**	73 (9%)	39 (10%)	14 (NA)		3,771 (11%)	706 (6%)
**2010 GDP per capita (USD)**	14,300	19,000	15,900	11,500	6,700	47,400

*Source*: World Health Organization (WHO). Global Health Observatory Data Repository. Found at http://www.who.int/tb/search/en/index.html. 2009.

The fall of the Soviet Union left Latvia’s health care system in disarray. TB diagnosis was disorganized and treatment haphazard. Treatment interruption, largely due to erratic drug supply, was one of the main drivers of the rise of drug-resistant tuberculosis during the unsteady 1990s [18]. High rates of MDR-TB identified 1994 prompted subsequent adoption of the DOTS-Plus strategy, with systematic response to drug resistance becoming standard [19]. As a result, Latvia has seen declining incidence and prevalence of MDR-TB [19,20]. We compared treatment costs and outcomes among MDR-TB and non MDR-TB patients in Latvia to partially identify the value of these investments.

## Methods

We retrospectively compared patients with identified MDR-TB to those with non MDR-TB to identify differences in health system cost stratified by patient outcomes. Latvia’s national tuberculosis control program, the *Nacionala Tuberkulozes Apkarošanas Programma* (NTAP) treats tuberculosis patients and provides other public health protections as well; the NTAP and its activities are well described elsewhere [18,19]. We obtained study data from NTAP’s national tuberculosis registry and patient medical records.

Study subjects received all tuberculosis treatment through Latvia’s TB control program. Records for all non-imprisoned, tuberculosis patients aged >=18 with treatment start dates during calendar year 2002 were eligible for inclusion. From these, we randomly selected every second MDR-TB patient and every 13^th^ non MDR-TB patient from the TB registry, starting from a random set point. These were included where basic and comparable data points were available; where such information was insufficient, we selected the next eligible subject from the registry.

We trained public health clinic staff within the NTAP to abstract clinical and outcome data from patient records using a standard methodology and data collection instrument. Demographic, clinical, and health system utilization data were abstracted. These included gender, age, geographic location, treatment delivery type, duration, and current treatment outcomes. Outcome measures were identified from the clinical impressions reflected in the patient records and were aggregated into three categories--cured, death, or not cured (included treatment failure and default). Patients with a history of prior treatment were also identified. Health system utilization data abstracted included duration of anti-tuberculosis treatment in days; the type and number of outpatient clinical encounters; duration of inpatient treatment; and the other medical supplies, services, or procedures consumed during anti-tuberculosis therapy, such as anti-tuberculosis drugs, laboratory tests, and imaging services. Susceptibility testing in Latvia is standard during diagnosis, and we identified resistance on that basis [20]. Data on extensively drug resistant (XDR) TB and quinolone resistance was not captured.

Cost data were obtained by study staff from clinic and other administrative records. Treatment cost was measured as a proportional share of the total annualized cost for all infrastructure, wages, utilities, supplies, and other goods and services consumed by inpatient and outpatient services. Outpatient service costs were denominated by encounter, e.g. the cost of one directly observed therapy visit for one patient. Inpatient care cost was denominated by patient day, and was estimated in a similar fashion to costs for outpatient care. Where staff, facilities, or other resources were shared between tuberculosis and other care, the cost of tuberculosis care was estimated based on the proportion of the resource consumed by tuberculosis care. For instance, the cost of facility infrastructure assigned to tuberculosis care was estimated as a function of the physical area used in that care. Costs were converted from the Lat to 2002 U.S. dollars (USD) for analysis and report.

Estimates of cost and outcomes are limited to those incurred by the public health system during the course of tuberculosis treatment. We did not attempt to estimate the economic costs or value associated with personal health losses or gains, such as acute illness, death, or cure. We did not estimate societal or other nonhealth system costs such as wage or other personal losses to a patient during treatment. Cost estimations that occur during the course of treatment are near term, representing periods of generally two years or less, and we did not discount or adjust these for time.

We used multivariate regression analysis to identify healthcare cost and utilization patterns associated with a diagnosis of MDR-TB. Negative binomial regression was used to model utilization outcomes (number of hospital days, clinic visits, specialist visits, x-rays and c computed tomography (CT)) adjusting for vital status, sex, age, and cured status. Linear regression analysis was used to examine inpatient, outpatient, prescription medication and overall costs in USD adjusting for type of TB infection, sex, age, and outcome. Robust standard errors are reported and all p-values were two-tailed. A p-value < 0.05 was considered statistically significant. All data, cost, and outcome measures are reported in the aggregate, and no personal or patient identifiers were collected, retained or reported. This program evaluation was determined to be nonhuman subject’s research by both the Office of the Associate Director for Science, U.S. Centers for Disease Control and Prevention, and by the University of North Texas Health Science Center’s Office for the Protection of Human Subjects; these determinations were provided, reviewed, and approved by program personnel within the NTAP.

## Results

We analyzed NTAP data from 92 MDR-TB and 54 non MDR patients beginning treatment in 2002. Patients with MDR-TB were more commonly male (78% vs. 63%, p-value=.05), middle aged (89% vs. 74%, aged 25–64, p-value=.019), and enjoyed lower cure rates (71% vs. 91%, p-value=.006) relative to those without (Table 2). Most 67% (61/92) MDR-TB patients had recorded prior tuberculosis treatment. No non MDR TB patients in our sample had prior tuberculosis treatment history (Table 2).

**Table 2:** Characteristics of non MDR and and MDR-TB patients, healthcare utilization, and cost (standard errors in parentheses), Latvia 2002

	**MDR-TB (n=9)**	**Non MDR-TB (n=54)**	**p-value**
Vital status, %			
Dead	6.6	3.7	.461
Alive	93.4	96.3	.461
Age, years	44.1 (1.3)	42.6 (2.5)	.558
Age group, %			
<25	6.6	14.8	.105
25-64	89.0	74.1	.019
65 or over	4.4	11.1	.123
Sex, %			
Male	78.0	63.0	.050
Female	22.0	37.0	.050
Treatment success, %			
Cured	71.4	90.7	.006
Not cured	28.6	9.3	.006
Previous treatment, # (%)			
None	31 (33%)	54 (100%)	<.001
First-line	55 (60%)	0	<.001
Second-line	6 (7%)	0	<.001
Hospital days, #	481.4 (25.0)	114.9 (12.6)	<.001
Clinic visits, #	73.7 (13.4)	70.4 (9.2)	.859
Specialist visits, #	11.0 (1.3)	5.1 (.6)	<.001
Sputum smears, #	35.9 (1.3)	12.9 (.7)	<.001
Sputum cultures,#	33.9 (1.4)	10.4 (.6)	<.001
X-rays, #	14.1 (.8)	6.4 (.5)	<.001
CT, #	1.0 (.1)	.5 (.1)	.011
Health care costs, 2002 USD $			
Inpatient	$25,004 (1,301)	$6,904 (759)	<.001
Outpatient	$1,672 (303)	$1,443 (188)	.587
Prescription medication	$2,146 (115)	$601 (3)	<.001
Total treatment	$28,821 (1,245)	$8,408 (649)	<.001

Patients with MDR-TB consumed more specialist visits, pharmaceuticals, laboratory services, x-rays, CT scans, and inpatient days than those with non MDR-TB, with average treatment cost 4 times greater (mean = 28,821 vs. 8,408 USD, p-value<.001) (Table 2). Outpatient care costs were not significantly different, but MDR-TB patients averaged almost an entire year longer as hospital inpatients than non MDR-TB patients (mean = 481 vs. 115 days, p-value<.001) at higher average cost (mean = 25,000 vs. 6,900 USD, p-value<.001). Medication cost for MDR-TB patients averaged 35 times (2,146 vs. 61 USD, p-value<.001) that of others.

Table 3 presents results from multivariate regression analysis of healthcare costs adjusting for type of TB infection, sex, age, and outcome. Healthcare costs included inpatient, outpatient, prescription medication and overall costs. Patients with MDR-TB were predicted to incur an additional 18,729 USD in inpatient treatment costs and 2,199 USD additional prescription medication costs compared to non MDR TB patients.

**Table 3:** Multivariate regression adjusted total cost of treatment by provider setting, 2002 $ USD (N=145)

	**Inpatient**	**Outpatient**	**Prescription medication**	**Overall**
Type of infection				
Non MDR-TB	Ref.	Ref.	Ref.	Ref.
MDR-TB	$18,729 (15,563, 21,893) ***	$592 (−192, 1,375)	$2,199 (1,956, 2,442)***	$21,520 (18,751, 24,288)***
Sex				
Female	Ref.	Ref.	Ref.	Ref.
Male	$5,002 (1,362, 8,641) **	-$834 (1,859, 191)	$228 (−41, 497)	$4,395 (1,358, 7,433)**
Age group				
<25	Ref.	Ref.	Ref.	Ref.
25-64	$4,310 (−1,294, 9,914)	-$436 (−1,781, 909)	$224 (−214, 661)	$4,097 (−987, 9,181)
>=65	$6,244 (−993, 13,480)	-$1,233 (−2,666, 199)	30 (−517, 577)	$5,040 (−1,738, 11,818)
Outcome of treatment				
Not cured	Ref.	Ref.	Ref.	Ref.
Cured	$7,707 (3,044, 12,371)***	$1,305 (328, 2,282) **	$923 (530, 1,315)***	$9,935 (5,370, 14,500)***
Vital status				
Alive	Ref.	Ref.	Ref.	Ref.
Dead	-$4,070 (−12,424, 4,283)	-$124 (−1,105, 857)	-$65 (−919, 788)	-$4,259 (−13,215, 4,696)
R^2^	.51	.10	.67	.63
N	145	145	145	145

* denotes p<.05; **, p<.01; ***, p<.001.

Multivariate negative binomial regression was used to examine healthcare utilization adjusting for sex, age and cured status. Estimated number of hospital days, clinic visits, specialist visits, X-rays, and CT for MDR and non MDR-TB patients adjusting for confounding factors are provided in Table 4. MDR TB patients are predicted to have spent one extra year in inpatient treatment compared to non MDR patients (475.3 vs. 110.2, p-value<.001). Similarly, MDR TB involved a higher number of specialist visits (11.0 vs. 4.2, p-value<.001), x-rays (13.9 vs. 6.4, p-value<.003) and CT (1.0 vs. .5, p-value=.003) than non MDR-TB.

**Table 4:** Adjusted health care utilization by MDR-TB diagnosis (n=145)*

	**Non MDR (n=54) Mean (CI)**	**MDR (n=92) mean (CI)**	**p-value of difference**
Hospital days, #	110.2 (86.1, 134.4)	475.3 (427.9, 522.6)	<.001
Clinic visits, #	53.3 (35.3, 71.3)	67.9 (42.5, 93.3)	.357
Specialist visits, #	4.3 (3.3, 5.4)	11.0 (8.6, 13.3)	<.001
X-rays, #	6.4 (5.4, 7.4)	13.9 (12.4, 15.5)	<.001
CT, #	0.5 (0.4, 0.7)	1.0 (.8, 1.2)	.003

* Negative binomial regression adjusts for vital status, sex, age and cured status. Chi-square test used to calculate p-values.

There was not a significant increase in clinic visits for MDR compared to non MDR TB patients, however. This may be explained by longer hospitalizations for MDR patients.

## Discussion

The growing presence of MDR-TB in Latvia presented clinical, administrative, and policy challenges related to its disproportionate impact on patient health and on health system costs. We analyzed healthcare, cost and health outcomes among Latvian TB patients to identify their interaction with treatment failure and its consequences. Our findings suggest the value of public investments that prioritize, sustain and enhance effective efforts against tuberculosis.

Prevention is a cornerstone of sustained and consistent public health infrastructure [20-24]. Drug resistance in TB patients is preventable, and begins with prompt and adequate treatment of susceptible *M. tuberculosis*. Even so, we found indications of treatment failure in our sample contributing to primary and acquired resistance. Prior treatment with first line and second line drugs was reported for 60% and 7% of study subjects with currently diagnosed MDR-TB. In contrast, there was no record of prior TB treatment for any of the non MDR-TB patients, who enjoyed better health outcomes and were treated at less than 1/3 the cost.

Multivariate linear regression revealed drug resistance as a robust predictor for the total cost for TB treatment and predicts where those costs fall most heavily. We found medication for treatment of MDR-TB medications to be the single largest contributor to its excess cost. The average duration of MDR-TB treatment among our cohort was almost twice that of non MDR-TB patients. Considering partial and incomplete treatments due to death, failure, and loss to follow-up, our data indicated that MDR-TB treatment averaged 293 days per patient—more than twice as long as the 147 days of treatment for the average non MDR-TB patient (results not shown). Lengthy treatment of MDR-TB consumed greater quantities of medication relative to that of other patients, at a much higher cost per dose for that medication.

These finding suggest a tangible value to tuberculosis control infrastructure that achieves treatment adherence and completion and controls development of resistance [25-28]. Our sample represents roughly 50% of Latvia’s MDR-TB incidence during our study period; we found most (61 of 92) of these had prior TB treatment. Had original treatment been sufficiently effective to prevent progression to MDR-TB, over $1,313,000 in health system savings—as well as health benefits from preventing such serious illness--might have been realized. Estimates based on our findings suggest every 10% reduction in multidrug-resistance nationwide would avert costs greater than $250,000 USD annually.

This analysis has limitations. Although data represents approximately one half of patients identified during 2002 with MDR-TB and about 7.5% of those with non MDR-TB, our sample size remained relatively small and lacked sufficient power to identify significant differences in more finely stratified analyses. To preserve analytical power, we aggregated the treatment outcomes in broader categories; however reported cure rates for newly incident TB in 2002 remained consistent with those reported elsewhere [29]. Case finding in Latvia remains passive, as persons with tuberculosis become known only after seeking care, often with more advanced or debilitating illness [19]. It is plausible that ascertainment bias among the non MDR-TB over-represents patients with more severe and costly illness, as they were known to the healthcare system through their in- and outpatient medical care. Although such sampling bias may overestimate the cost of care and the severity of outcomes, this still allows the most conservative estimate of these impacts and changes only the magnitude, not the direction, of our findings. Duration of outpatient care was not recorded, and assessment of treatment success was dichotomous—success or not. We were therefore unable to evaluate the effects on cost and outcome of partial treatments, to identify the rate of medical system utilization as a function of time, or to denominate outpatient care as a cost per day. Our data was not longitudinal; hence allowing only point estimates. We did not have sufficient sample size to allow a robust stratified analysis. It is likely that we underestimate the actual value of successful control of MDR-TB, and may undervalue the potential benefits of prevention. We reported only treatment costs incurred by the health system. A more broad estimate, such as from a societal perspective, might include many elements we did not. These might include measures of individual health losses from morbidity and mortality among patients or their contacts; other public health infection control activities such as contact tracing; or of resources consumed outside of the public health system [13,14,30-32]. It is also important to note that in 2002, Latvia procured second-line drugs through a mechanism of the Green Light Committee that reduced cost to the program [33]. As such, the data regarding drug cost of MDR-TB maybe artificially low.

These limitations do not compromise our conclusions, however. We were able to identify patterns that suggest an association between treatment or program failure and multi-drug resistance. We identified the cost of cure for TB patients, and we identified the cost and some clinical outcome differentials between non MDR and MDR-TB patient care by major categories. We estimated the opportunity costs associated with MDR-TB from the public health setting perspective, and by doing so allow a plausible estimate of the lower bound value of reduced treatment or program failure.

Political boundaries are no protection from the dangers of MDR-TB to an international community. The effective control and prevention of acquired resistance represents an opportunity for health preservation well beyond the borders of Latvia. Global health protections require a consistent and united tuberculosis control infrastructure, however, and the value of these may not be apparent to health policy decision makers. We have clearly and conservatively identified the opportunity costs associated with the public treatment of MDR-TB patients in Latvia. This may allow a more clear judgment of the value of its control, and may be a critical component in the public fight against this dangerous disease.

## Competing interests

The authors declare that they have no competing interests.

## Authors’ contributions

VL, TH, KC, AC, PM, and VR collaborated to conceive the study and to direct data collection and management. TM and FW designed and conducted the analysis and led its interpretation. TM led the drafting and refinement of the manuscript with the participation of FW, VL, TH, KC, AC, PM, and VR. All authors read and approved the final manuscript.
